# Synovial chondromatosis of the elbow in a child

**DOI:** 10.4103/0019-5413.77141

**Published:** 2011

**Authors:** Rishi Narasimhan, Stuart Kennedy, Sandeep Tewari, Deeksha Dhingra, Ibrahim Zardawi

**Affiliations:** Orthopaedic Surgery Department Manning Base Hospital, High Street, Taree, NSW 2430, Australia

**Keywords:** Child, painful elbow, synovial chondromatosis, trauma

## Abstract

Synovial chondromatosis is cartilaginous metaplasia of mesenchymal remnants of synovial tissue of the joints. Its main characteristic is the formation of cartilaginous nodules in the synovium and inside the articular space (loose bodies). It usually presents between the third and fifth decades and is rare in children. It presents as a mono-articular pathology affecting large joints such as the knee, hip, and elbow. The main symptoms are pain, swelling, and limitation of movements in the affected joint. Diagnosis is made by panoramic radiographs, computed tomography scan, and mainly magnetic resonance imaging and on surgery. The authors describe of synovial chondromatosis presenting in the elbow of an 11 year-old girl which is unreported to the best of our knowledge.

## INTRODUCTION

Synovial chondromatosis is cartilaginous metaplasia of mesenchymal remnants of synovial tissue.^1^,^2^ Simultaneous production of multiple discreet lesions implies a reaction of residual embryonal cells with suggestion of possible neoplastic etiology. Traumatism, parafunctions, and infections along with Fibroblast growth factors (FGF 9) have been implicated in the pathogenesis.^1^,^3^,^4^

Multiple discreet nodules presenting as intraarticular loose bodies is the hallmark. It is rare in children and usually presents between third and fifth decades being twice as common in males as compared to females.^1^ The synovial chondromatosis is not described in a paediatric patient, hence this report.

## CASE REPORT

The authors have obtained informed written consent from the patient’s guardian for print and electronic publication of the case report.

**Figure 1:**
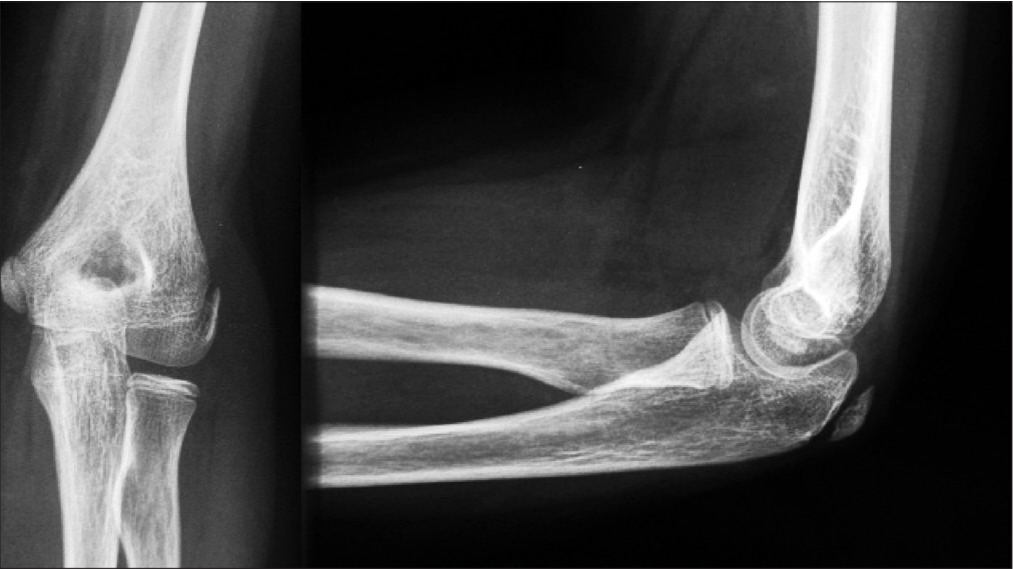
Initial X-rays of the left elbow showing only a dense lateral condyle

An 11-year-old girl presented with persistent left elbow pain 2 months after a roller skating accident. Initial examination revealed tenderness over the medial, lateral epicondyles, and the radial head. She was unable to touch the shoulder tip and her elbow range of motion was between 10 and 140°. Pronation and supination were unrestricted. Initial X-rays being normal [Figure 1] and activity modification was advised.

**Figure 2:**
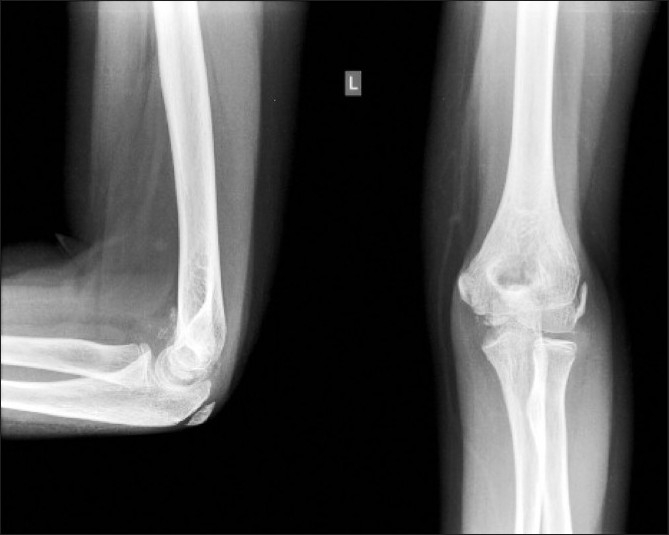
X-rays at three month follow up showing bone formation anterior and posterior to the distal humerus, suggesting a missed supracondylar fracture, capsular calcification and or loose bodies

At her second follow-up, a month later additional tenderness was noted over the olecranon. Comparison X-rays of both the elbows were normal, and expectant management was continued. Pain at rest and with resisted movements was noticed at the 3-month review. Repeat X-rays [Figure 2] showed bone formation anterior and posterior to the humerus suggesting a missed supracondylar fracture with the presence of capsular calcification or loose bodies.

**Figure 3:**
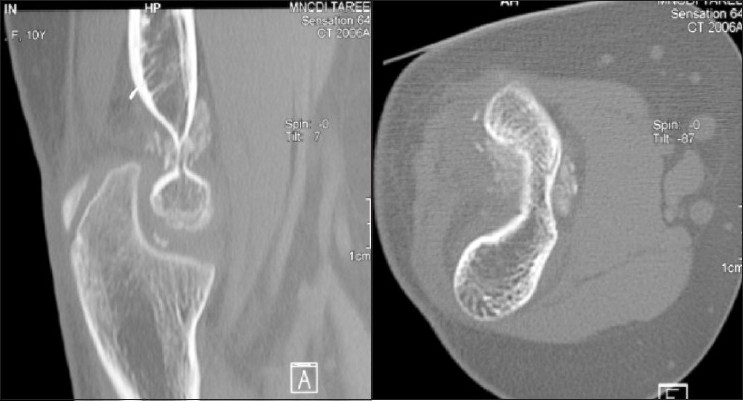
CT scan of the left elbow showing demineralisation of bones in distal humerus, radius and ulna, with disrupted appearance of trochlea. Bone fragments are seen both anteriorly and posteriorly

**Figure 4:**
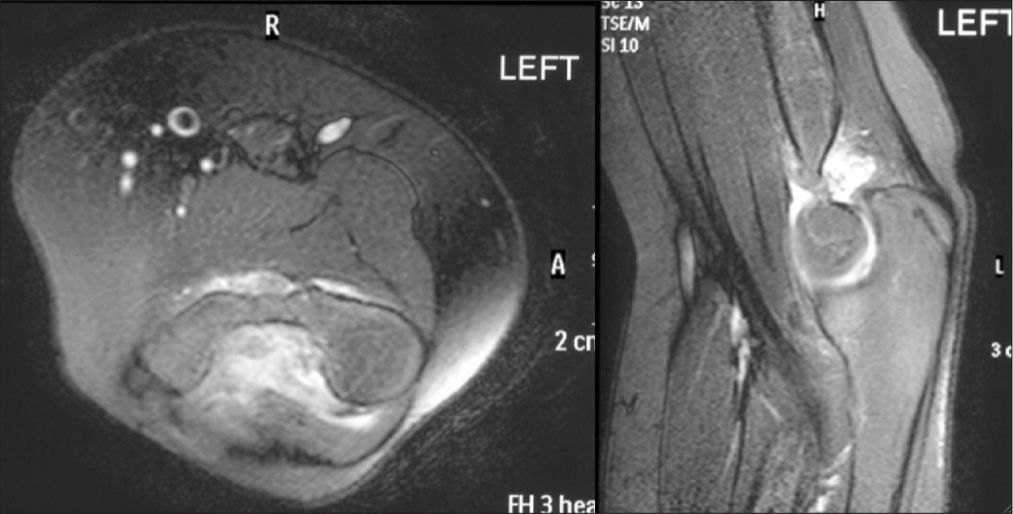
MRI scan of the left elbow showing joint effusion without evidence of bony injury. Loose bodies (osteochondral fragments) seen between trochlea and olecranon in saggital images, donor site was not identified. Ligaments, common flexor and extensor origins appeared intact

Computed tomography (CT) scan [Figure 3] of the elbow showed bone fragments anterior and posterior to the distal humerus while magnetic resonance imaging (MRI) scan [Figure 4] ascertained the presence of loose bodies in the elbow.

**Figure 5:**
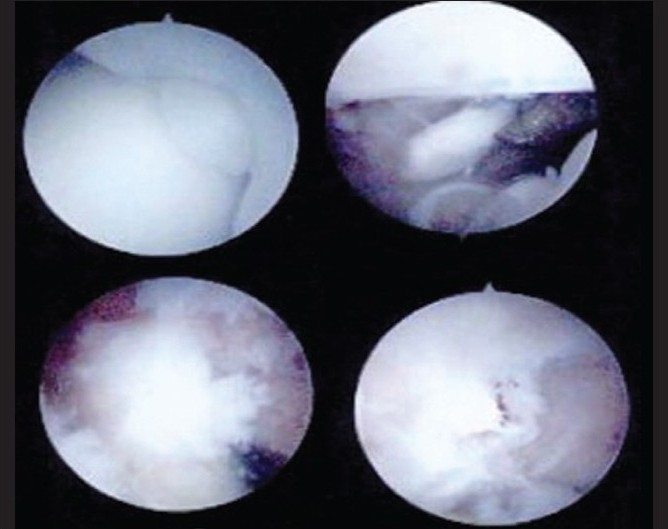
Initial arthroscopy images showing absence of synovitis with evidence of multiple loose bodies in the anterior and posterior compartments

The patient then had a diagnostic elbow arthroscopy which showed no evidence of synovitis or articular cartilage damage. Multiple loose bodies (two in anterior and five in the posterior compartment ranging in size from 2 to 10 mm) were visualized [Figure 5]. The loose bodies were extricated along with enveloping synovial tissue and sent for histopathology.

**Figure 6:**
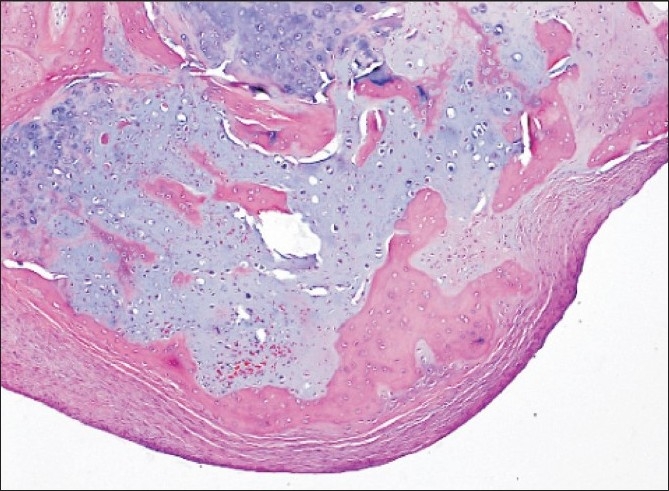
Osteochondral loose body, consisting mainly of central cartilaginous material with focal endochondral ossification and covered with a layer of fibrotic synovial membrane. Attenuated synovial cells are present on the surface (Haematoxylin and Eosin stained section, original magnification ×5 objective)

Histopathology [Figure 6] revealed circumscribed lobulated nodules of osteocartilaginous tissue with the stroma consisting of cellular fibroblastic tissue. Some fragments were covered with synovial tissue. There was no evidence of infection or malignancy in the specimen, and this was consistent with the appearance of synovial chondromatosis.

She regained full range of motion and was pain free following her arthroscopy. However, she presented a year after being discharged from the orthopadic service with recurrence of elbow pain along with episodic catching. Physical examination revealed posteromedial elbow pain worse with elbow extension. Her range of motion was slightly restricted (10–140°) but as before pronation and supination of the forearm were normal.

**Figure 7:**
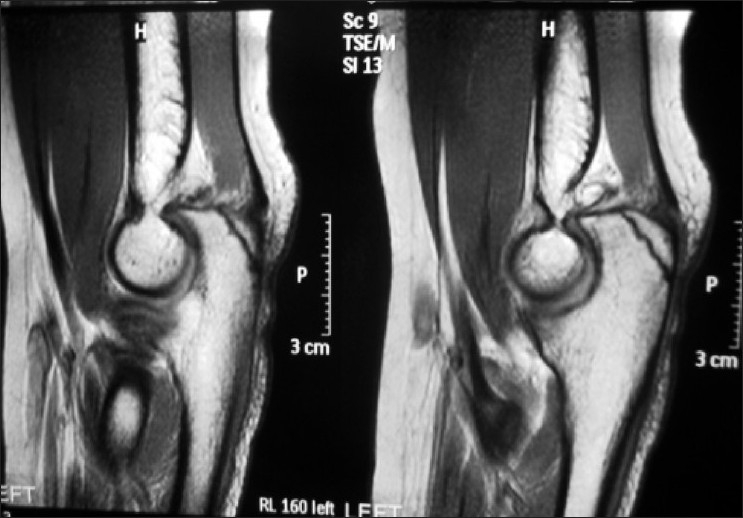
MRI scan showing presence of a loose body in the posterior compartment with a possible osteochondral defect in the dorsal articular surface of capitellum

**Figure 8:**
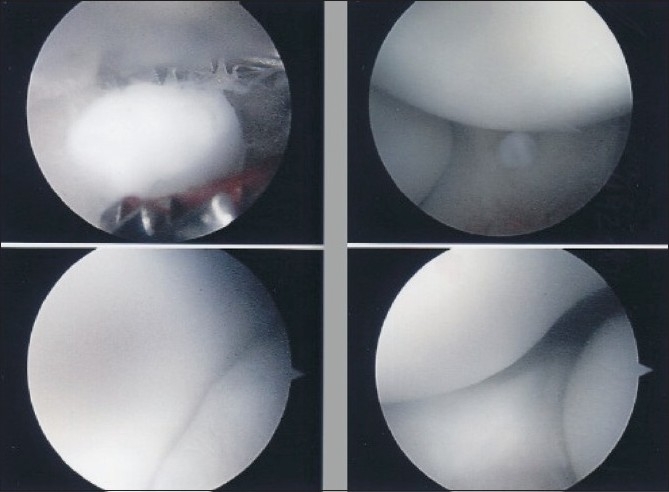
Repeat arthroscopy showing a small osteochondral body in the anterior compartment which was adherent to the synovium. The posterior had a large free floating loose body. There was no evidence of synovitis or articular cartilage damage in either compartment

**Figure 9:**
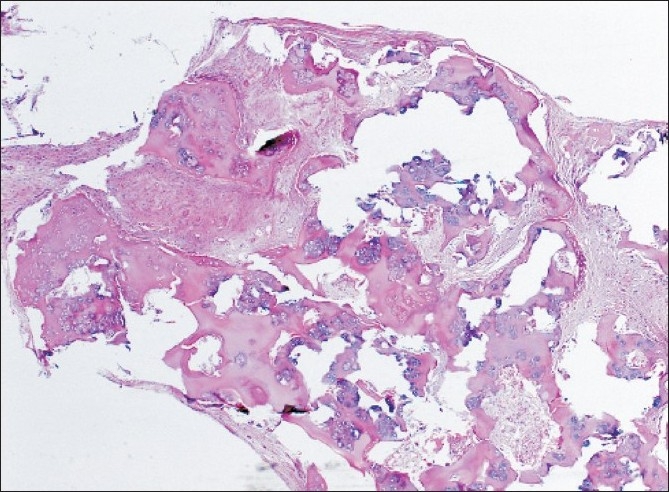
Lobulated osteochondral loose body, consisting predominantly of cartilaginous with focal endochondral ossification and fibrous marrow spaces (Hematoxylin and Eosin stained section, original magnification ×2.5 objective)

Elbow X-ray was normal, but MRI scan [Figure 7] showed a loose body in the posterior radiocapitellar joint along with an osteochondral defect in the dorsal capitellum. No loose bodies were seen in the olecranon and coronoid fossa. She was taken for her second elbow arthroscopy almost 2 years after her index operation. The posterior compartment revealed a large loose body (5–6 mm) whereas the anterior compartment had a small (2 mm) osteochondral body still attached to the synovium by a peduncle [Figure 8]. Both of these were removed easily, and synovectomy was performed in both anterior and posterior compartments. The specimens were sent for histopathology which was consistent with synovial chondromatosis without any evidence of infection or malignancy [Figure 9].

At the last follow-up 2 weeks after surgery, the patient reported complete resolution of pain and catching and her elbow range of motion was normal.

## DISCUSSION

The synovial chondromatosis affects mainly large joints such as the knee, hip, elbow, and temporomandibular joint (TMJ). Extraarticular synovial chondromatosis has been reported in the hands, feet, wrist, knee, elbow, finger, and iliopsoas bursa. It commonly presents as monoarticular pathology with pain and swelling in the joint along with the presence of intracapsular masses. Lesions can invade the joint capsule and present as extraarticular masses.^1^,^3^ Malignant degeneration into chondrosarcoma has been reported.^3^ Ultrastructural differentiation between the synovial chondromatosis and synovial chondrosarcoma is difficult with the permeative growth pattern strongly suggesting malignancy and the importance of seeking a second opinion in case of doubt is highly stressed.^3^,^5^

The disease has three phases. The early phase is of active synovitis without loose bodies. The second phase shows nodular synovitis along with loose bodies, and phase three is characterized by the presence of loose bodies with resolution of synovitis.^6^,^7^ The current patient had stage three disease.

Two forms of the disease have been described, the primary form presenting insidiously with joint effusion, palpable loose bodies, crepitus and loss of ROM and/or peripheral neuropathy. Clicking and locking joints are commonly noted. The primary form is progressive leading to severe degenerative arthritis, with possibility of recurrence after treatment. Secondary forms of the disease are associated with slowly progressive degenerative joint disease, trauma, inflammatory and noninflammatory arthropathies, and neurologic disease. Secondary cases show no histological atypia, and grow in a more organised way and tend not to recur after excision thus having a low risk of developing malignant degeneration.^1^

Initial radiographs may show loose bodies in the joint with erosion of articular cartilage, but can underestimate the size of the lesions. CT scans can delineate the loose bodies better. MRI can define the extent of the lesion, identify involvement of other structures such as adjacent marrow, soft tissues, and neurovascular structures apart from differentiating it from other synovial lesion which showing typical MRI features.^1^,^7^

Treatment of synovial chondromatosis entails synovectomy with removal of the loose bodies in the presence of active synovitis.^1^,^6^,^8^ The recurrence rate after surgery ranges from 3.2% to 22.2%.^5^ Radiotherapy is beneficial for recurrent lesions and inhibition of FGF-9 has been suggested as a nonoperative method of treating primary synovial chondromatosis.^4^,^9^

## CONCLUSION

Painful limitation of movement may be more than just post-traumatic stiffness. MRI is the preferred modality in differentiation of synovial lesions.^3^,^8^ Biopsy usually confirms the diagnosis. Patients need to be told that synovial chondromatosis can recur and follow-up is necessary. Malignant change is extremely rare, but is documented.
