# CORRIGENDUM

**Published:** 2011

**Indian J Orthop March 2011; Vol 45; Issue 2** **Title: Synovial chondromatosis of the elbow in a child** **Page 181; Introduction : Para 2 : Line 4** **The synovial chondromatosis is not described in a paediatric patient, hence this report** *Should read as* **The synovial chondromatosis treated by arthroscopy in a pediatric case is not described hence this report**
The error is regretted *- Editor, IJO*

